# The Impact and Mechanism of Action of *Peptostreptococcus anaerobius* on Chemotherapy Resistance in Human Colorectal Cancer

**DOI:** 10.5152/tjg.2024.24221

**Published:** 2024-10-01

**Authors:** Guangcai Li, Wenwen Li, Hongsheng Dai, Xialian He, Lihong Shi, Xiaoqian Zhang

**Affiliations:** 1 Department of Gastroenterology, Affiliated Hospital of Shandong Second Medical University, School of Clinical Medicine, Shandong Second Medical University, Weifang, China; 2 Clinical Research Center, Affiliated Hospital of Shandong Second Medical University, Weifang, China; 3 School of Rehabilitation Medicine, Shandong Second Medical University, Weifang, China

**Keywords:** Colorectal cancer, chemotherapy resistance, *Peptostreptococcus*, interleukin 23

## Abstract

**Background/Aims:**

*Peptostreptococcus anaerobius* plays an important role in the development of colorectal cancer, and previous studies by our group have demonstrated that *Peptostreptococcus anaerobius* promotes resistance to 5-Fu chemotherapy in animal models of colorectal cancer. In this study, the effects of *Peptostreptococcus anaerobius* on chemotherapy resistance in colorectal cancer and its possible mechanism of action were investigated from the clinical point of view.

**Materials and Methods:**

Patients were selected according to exclusion and inclusion criteria and divided into sensitive and chemotherapy groups (n = 20/group). Fecal samples were collected from the patients. The bacterial 16S rRNA genes in the samples were sequenced and the abundance and varieties in the fecal bacteria were compared between the 2 groups. Immunohistochemistry and Western blotting were used to assess interleukin 23 levels in tumor tissues.

**Results:**

Significantly elevated abundance of *Peptostreptococcus* was observed in fecal samples from chemoresistant colorectal cancer patients compared to those from chemosensitive individuals. Immunohistochemistry and Western blotting results showed that chemoresistant patients had higher levels of interleukin 23 relative to chemosensitive patients and the levels were positively associated with *Peptostreptococcus*.

**Conclusion:**

*Peptostreptococcus* may mediate the development of chemoresistant colorectal cancer by promoting the upregulation of interleukin 23. Efforts to target *Peptostreptococcus* thus have the potential to alter the prognosis of colorectal cancer patients.

**Main Points**

- *Peptostreptococcus* may be involved in chemotherapy resistance in human colorectal cancer (CRC).
- *Peptostreptococcus* upregulates interleukin-23 expression in the tumor microenvironment.
- Efforts to target *Peptostreptococcus *thus have the potential to alter the prognosis of CRC patients.

## Introduction

Colorectal cancer (CRC) incidence continues to rise annually, making it the third most common type of malignancy and the second deadliest form of cancer, contributing to a morbidity and mortality rate exceeding 10%.^1^ While 5-FU and oxaliplatin-based chemotherapeutic treatments have significantly improved prognostic outcomes for CRC patients,^2^ the emergence of chemoresistance contributes to poor prognostic outcomes for these patients. The development of resistance to medication is one of the primary causes of treatment failure, affecting about 90% of patients with metastatic disease.^3^ An understanding of the mechanisms underlying resistance is thus essential for the treatment of CRC. Various mechanisms have been proposed, including abnormalities in membrane transport proteins, impaired cellular metabolism, intra-tumor heterogeneity, and changes in metabolic enzymes.^4-6^ The roles played by the gastrointestinal microflora and the tumor microenvironment (TME) in this context have been the focus of increasing research interest in recent years.

The human gastrointestinal tract is colonized by an estimated 1 billion bacteria, comprising an immunologically active community that must be taken into consideration.^7^ CRC incidence and progression are often associated with intestinal dysbiosis, and changes in the abundance of certain bacteria can also contribute to CRC progression and patient prognosis.^8^ *Peptostreptococcus* has gradually emerged as an important mediator in this context, interacting with the TLR2 and TLR4 receptors expressed on colonocytes while promoting CRC development via modulating TME composition by MDSCs and TAM.^9,10^ Weiting Ge and Hanguang Hu et al reported that patients with high-risk CRC overexpress IL-23A and have higher levels of cancer-associated gut bacteria such as *Peptostreptococcus* and *Bacteroides fragilis*. It has been proposed that metabolites from these bacteria stimulate the production of interleukin-23 (IL-23) by MDSC.^11^ IL-23 is a cytokine with immunosuppressive activity that also plays an important role in treatment resistance. Resistance to cisplatin was observed in tumor cells from patients with laryngeal cancer; these cells overexpressed the IL-23 receptor (IL-23R) and STAT3 phosphorylation was found to be linked to cisplatin resistance.^12^ It has been observed that IL-23 promotes the epithelial-to-mesenchymal transition (EMT) during the development of esophageal and gastric cancer.^13,14^ The EMT is known to contribute to the development of chemoresistance by preventing apoptosis in tumor cells, as well as by increasing cell stemness.^15^ These findings point to a possible function for IL-23 in the development of chemoresistance, which contributes significantly to the poor prognosis of CRC.

A previous study by our group on an animal model of CRC suggested that *Peptostreptococcus* could reduce sensitivity to 5-Fu in CRC and thus promote chemoresistance.^16^ To date, there have been no investigations of the role of *Peptostreptococcus* in chemoresistance in human CRC. Here, it was hypothesized that *Peptostreptococcus* plays a role in chemoresistance in human CRC, and this was investigated in human patients from a clinical point of view. The findings provide a reference for studying the mechanisms underlying chemoresistance in CRC with the goal of improving patient clinical outcomes.

## Materials and Methods

### Patient Selection

This study enrolled CRC patients who underwent treatment with a combination of 5-FU and oxaliplatin and were admitted to the Affiliated Hospital of Shandong Second Medical University prior to 2019, for whom 5-year follow-up data were available. This study was performed in line with the principles of the Declaration of Helsinki. Approval was granted by the Medical Ethics Committee of the Affiliated Hospital of Shandong Second Medical University (approval no: wyfy-2023-ky-136, date: June 5, 2023).

Based on whether patients exhibited recurrent or metastatic disease within 5 years after surgery, patients were classified into chemosensitive and chemoresistant groups (n = 20/group). Inclusion criteria: (1) 18-65 years of age, (2) clinically and pathologically diagnosed with CRC, (3) free of other serious comorbid diseases, (4) free of any history of taking antibiotics, probiotics, glucocorticoids, immunosuppressants, or other drugs with the potential to alter the composition of the gastrointestinal microbiome within 6 months before the study, and (5) demonstrated good compliance and underwent follow-up. Exclusion criteria: (1) diagnosed with other gastrointestinal disorders with the potential to impact the microbiota, (2) had taken any drugs with the potential to adversely impact the microbiota within 6 months prior to study participation, (3) exhibited any severe comorbid diseases, or (4) had incomplete clinical information. Before samples were collected, patients were informed of the goals of the study and the sample collection process, and informed consent was obtained. Samples were then collected. Patients were monitored over the course of follow-up for evidence of metastatic or recurrent disease via imaging (chest and abdominal CT), gastroenteroscopy with biopsy, and telephone follow-up.

### Sample Collection and DNA Extraction

Patient fecal samples were collected in sterile boxes marked with relevant information and stored at −80°C. For patients meeting the inclusion criteria outlined above (n = 20/group), the TGuide S96 magnetic bead method soil/feces DNA extraction kit (DP812, Tiangen Biochemical Technology Co., Ltd.) was used to extract total bacterial gDNA based on provided instructions, after which an enzyme labeling instrument was used to quantify DNA concentration and purity. The V3-V4 hypervariable region of the bacterial 16S rRNA gene was amplified with the 338F (5’-ACTCCTACGGGGAGGCAGCA-3’) and 806R (5’-GGACTACHVGGGTWTCTAAT-3’) primer pair. A Quant-iT 1X dsDNA HS kit was used for the quantification of the resultant PCR products. A Novaseq 6000 instrument (Illumina) was then used for the PE250 sequencing of prepared samples, and species were analyzed with the Silva database.

### Immunohistochemistry

Clinicopathologic characteristics and 5-year follow-up data for all patients were obtained from the follow-up database, after which colorectal tumor tissue sections from chemosensitive and chemoresistant groups (n = 20/group) were selected for immunohistochemical (IHC) staining. Sections were deparaffinized, rehydrated, treated with citric acid antigen repair solution (pH 6.0), and incubated overnight with anti-IL-23 (Bioss, bs1193-R) at 4°C. After secondary incubation with HRP-labeled goat anti-rabbit IgG (GB23303, Servicebio), a DAB kit (G1212, Servicebio) was used for color development and hematoxylin was used for counterstaining. Six highly magnified (400×) fields of view per section were selected at random, and average optical density values for each section were computed in a semi-quantitative manner with ImageJ.

### Western Blotting

To detect IL-23 levels in tumor tissues, samples were frozen at −80°C, after which proteins were extracted using a standard approach, and protein levels in the resultant lysates were quantified through a BCA assay approach. Proteins were separated via 12.5% SDS-PAGE, transferred onto a PVDF membrane, and blots were blocked for 90 min with 5% non-fat milk prior to overnight incubation with an appropriate primary antibody (Bioss, bs1193-R) at 4°C. Following a 1 h incubation with an appropriate secondary antibody, protein bands were detected with a chemical gel luminescence system, and ImageJ was used for densitometric quantification. β-Actin expression was analyzed as a loading control.

### Statistical Analysis

Data were analyzed and graphed using the Statistical Package for the Social Sciences version 25.0 (IBM Corp., Armonk, NY, USA) and GraphPad Prism 8.0 (GraphPad Software). Normally distributed data were compared with independent samples *t*-tests, while non-parametric tests were used for all other data. Relationships between *Peptostreptococcus* copy numbers and IL-23 expression in patient samples were analyzed through Pearson’s correlation analysis. A test level of *α* = 0.05 and *P* < .05 were used to define statistical significance.

## Results

### Analyses of the Intestinal Flora Composition in Chemosensitive and Chemoresistant CRC Patients

**Figure 1.**
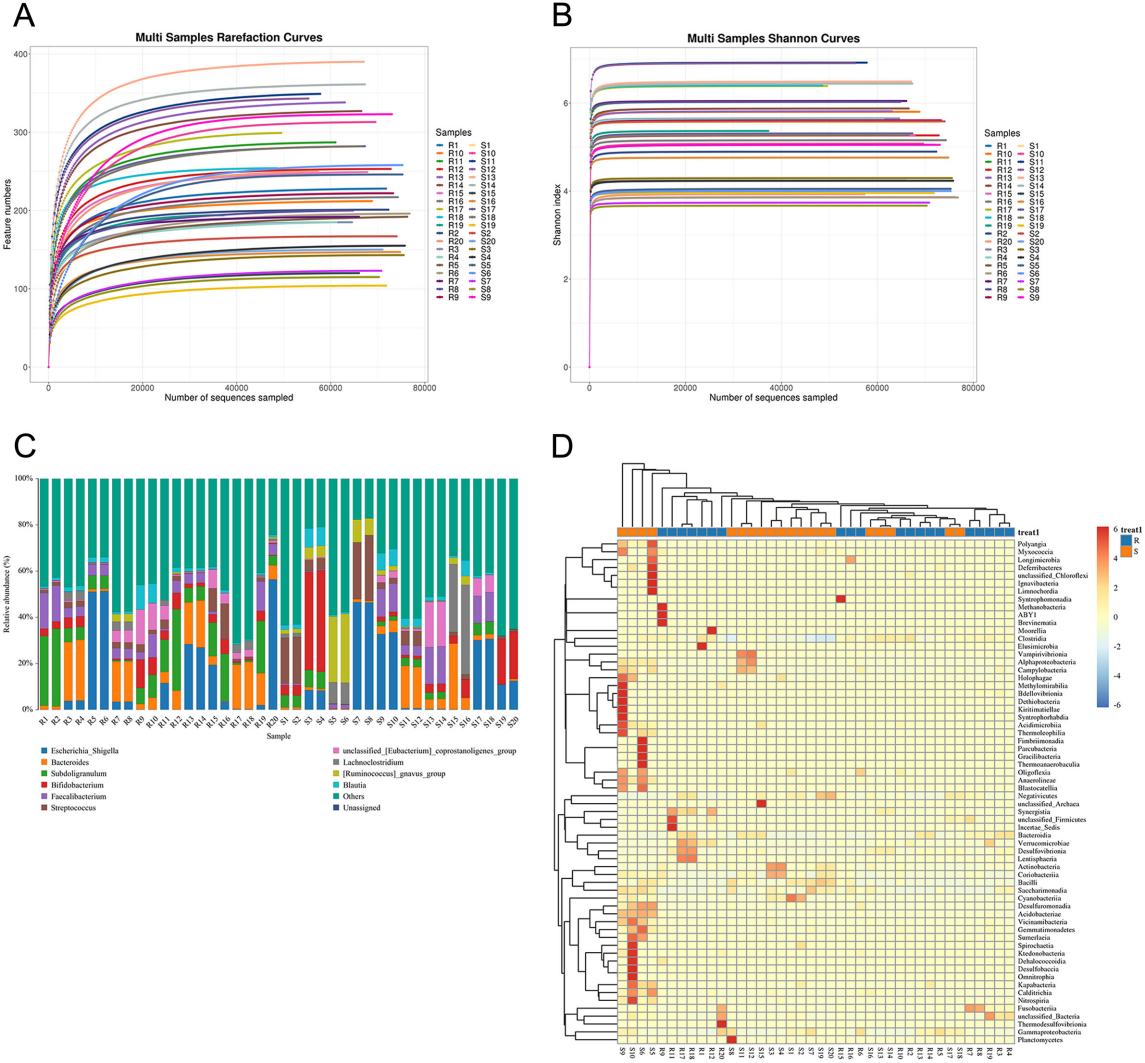
Differences in microbial abundance between the different samples. (A) Multi-sample rarefaction curves; (B) multi-sample Shannon curves; (C) differences in microbial abundance at the genus level between the samples; (D) heatmaps showing clustering of species abundance.

With increasing numbers of sequenced samples, the number of identified OTUs and the Shannon index curve increased before gradually beginning to level off (Figure 1A and 1B). This indicates that sufficient sequencing data was collected and that the number of characteristic species was not likely to increase further with additional sequencing, permitting subsequent analyses. Relative bacterial abundance at the genus level was compared between groups (Figure 1C), with the resultant clustering heat map highlighting the similarities and differences between these 2 patient groups (Figure 1D). Overall, clear differences in bacterial abundance were evident when comparing chemoresistant and chemosensitive patient fecal samples.

### Examination of Differences in Gastrointestinal Flora Diversity as a Function of Chemoresistance

**Figure 2.**
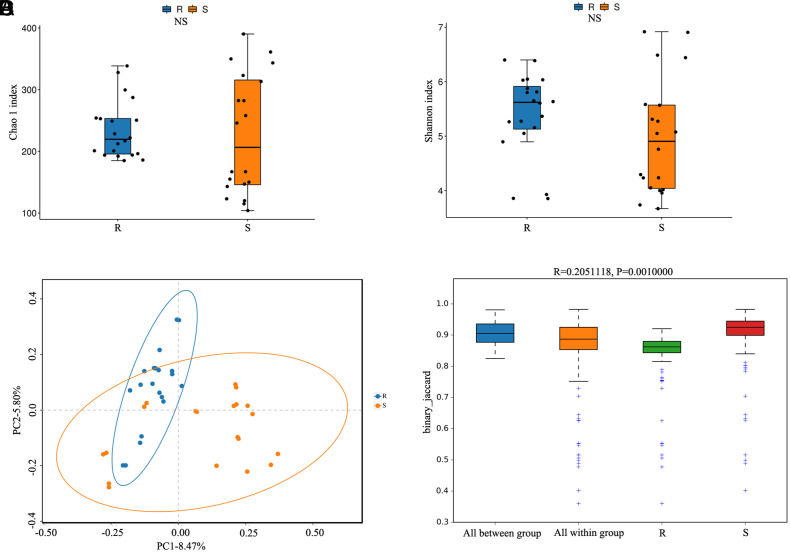
Differences in microbial diversity between the samples. (A) Chao1 index of identified OTUs; (B) Shannon index of identified OTUs; (C) Beta diversity comparison using PCoA analysis based on the Bray–Curtis distance matrix; (D) Statistical analysis of beta diversity (R, group; S, chemotherapy-sensitive group; between, between the 2 sample groups; within, within the samples; NS, non-significant; *P* > .05).

No significant differences in Chao1 and Shannon index values were noted between sample groups, indicating that bacterial species richness and diversity were similar in both groups (Figure 2A and 2B). A PCoA analysis was used for sample classification, highlighting differences in species diversity between groups such that these 2 sets of samples were clearly separated from one another (Figure 2C). ANOSIM analyses additionally revealed clear differences in β-diversity between samples from these groups, with between-group differences being greater than within-group differences such that this grouping strategy was considered meaningful (Figure 2D).

### *Peptostreptococcus* Abundance is Associated with Chemoresistance in CRC

A Metastats analysis revealed differences in relative bacterial abundance in both groups, with the abundance of *Peptostreptococcus* in the chemosensitive group being less than 0.2%. Marked intra-group differences in *Peptostreptococcus* abundance were evident, likely owing to individual differences. Even so, *Peptostreptococcus* abundance in chemoresistant patient samples was significantly increased compared to chemosensitive patient samples (*P* < .01).

### Detection of IL-23 by IHC Staining

IHC analyses of IL-23 expression in tumor tissue samples from both groups revealed its expression in the cytosol in both groups. Darker staining was evident in samples from chemoresistant patients relative to those from chemosensitive patients, with a significant increase in the mean optical density values for IL-23 in CRC patients with chemoresistant disease (*P* < .05).

### Detection of IL-23 by Western Blotting

Relative to tumors from chemosensitive CRC patients, those from chemoresistant patients exhibited significantly higher IL-23 expression as detected by Western blotting (*P* < .05).

### Correlations Between Relative *Peptostreptococcus* Abundance and IL-23 Expression

Pearson correlation analyses revealed that relative *Peptostreptococcus* abundance was significantly positively correlated with the expression of IL-23 as measured by Western blotting (*r *> 0.6, *P* < .05).

## Discussion

CRC incidence is continuing to rise annually, with over 1.9 million diagnoses and 935,000 deaths in 2020 alone, accounting for roughly 10% of all cancer-related deaths.^1^ The first-line adjuvant and palliative treatment of CRC patients generally centers around a chemotherapeutic regimen composed of 5-FU and oxaliplatin, which can contribute to the significant prolongation of patient survival with associated quality of life improvements.^2^ However, due to a small proportion of tumor cells (tumor stem cells) becoming resistant to the treatment, the number of patients presenting with recurrent or metastatic CRC is increasing, and at this stage, the 5-year survival rate of patients with advanced CRC in China is less than 20%.^17^ There is thus a pressing need to clarify the mechanisms responsible for the emergence of chemoresistance in CRC. Many researchers currently believe that such drug resistance is the result of cellular metabolic disorders, abnormal membrane transporter protein activity, and changes in the TME.^18,19^ A growing body of work has also emphasized the importance of gastrointestinal flora in this context. Indeed, chemoresistant patients have been shown to exhibit increased Fusobacterium abundance, promoting the emergence of chemoresistance as a result of the regulation of autophagic activity.^20^ Fusobacterium-derived succinic acid can inhibit the cGAS-IFN-β pathway, thereby supporting CRC tumor resistance to immunotherapeutic interventions. These results emphasize the importance of gastrointestinal microbes in CRC resistance to drug treatment. In addition to *Fusobacterium*, *Peptostreptococcus *enrichment is evident in the intestines of chemoresistant CRC patients,^20^ and represents a potential diagnostic biomarker of the disease.^21^ The role that *Peptostreptococcus* plays in the emergence of such chemoresistance, however, remains uncertain.

**Figure 3.**
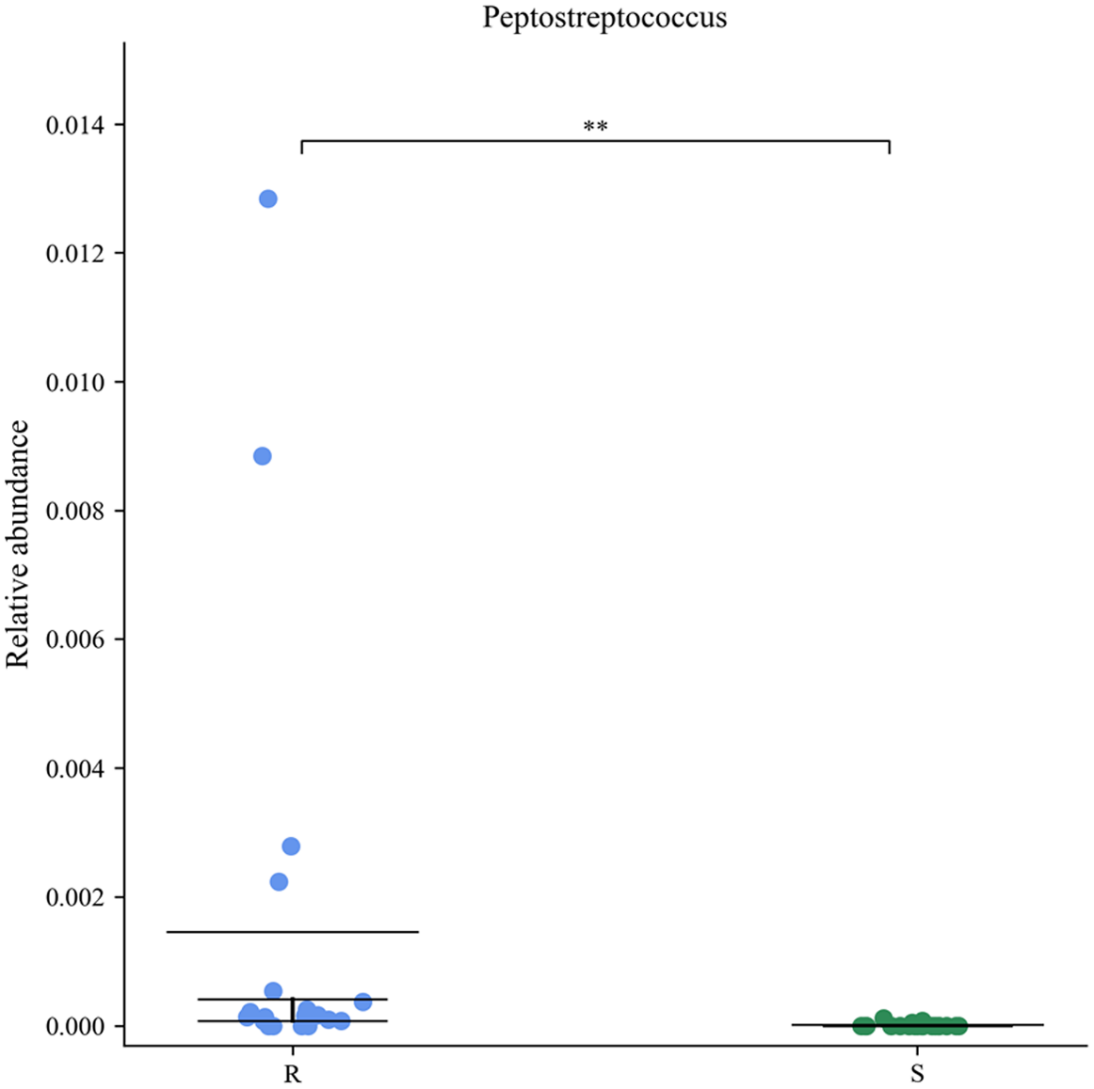
Differences in the relative abundance of *Peptostreptococcus* by Metastats (R, chemotherapy-resistant group; S, chemotherapy-sensitive group; **P < .01).

In a previous study on an animal model of CRC, our group discovered that* Peptostreptococcus* plays a role in chemoresistance in CRC.^16^ However, clinical studies on the association were not performed. As described above, *Peptostreptococcus* appears to be closely associated with CRC development. Here, 20 patients with CRC who satisfied the inclusion and exclusion criteria provided fecal samples that were then analyzed by high-throughput sequencing. This showed that there was no discernible variation in the α diversity of the intestinal flora between patients in the sensitive and the chemotherapy-resistant groups (Figure 2A and 2B). On the other hand, differences in the relative abundance of the typical flora were observed (Figure 1C). The dynamic balance of the gut flora can be affected by physiological changes in the host. Previous research found that experimental animals treated with 5-Fu showed reductions in both the number and variety of microorganisms in the gut microbiota.^22,23^ The present study found that the gut flora of the patients in both groups was impacted by the chemotherapy medications they received and there was thus no significant difference between them in terms of species richness. A possible explanation for this is that the dominant flora were able to proliferate because the flora of the drug-resistant hosts had adapted to the chemotherapy medication. The distinctive gut flora changed as a result of immune modulation and metabolite alterations. Analysis using Metastats showed that the relative abundance of anaerobic digestive streptococci was significantly higher in samples from the drug-resistant group than in those from the drug-sensitive group (Figure 3, *P* < .01). These findings confirm our earlier animal investigations as well as the results of Yu et al.^20^ *Peptostreptococcus* and CRC have been linked in previous research. Nevertheless, despite the evidence of the involvement of *Peptostreptococcus* in CRC carcinogenesis, its function in chemoresistance requires further investigation.

TAMs can be remodeled by *Proteostreptococcus*,^9^ and chemotherapy resistance and tumor development are actively facilitated by the TME.^24,25^ The cytokine IL-23 is a crucial part of the TME and has drawn specific attention in studies of tumors.^26^ It is able to control the function of other cells in the TME, modulate the host immune response, and directly affect the behavior of both precancerous cells and malignant tumors. Clinical studies have shown that increased levels of IL-23 are correlated with CRC histological grading as well as with the levels of vascular endothelial growth factor.^27,28^ Additionally, as seen in gastric and esophageal cancer, IL-23 promotes the EMT via the Wnt/β-connexin and STAT3 pathways.^13,14^ It has even been proposed that evidence of EMT activation in tumor biopsies can be utilized as a prognostic marker for treatment-sensitive or medication-resistant patient subgroups, as the EMT increases cell stemness and reduces apoptosis, both of which contribute to the development of drug resistance.^29,30^ These data point to a possible involvement of IL-23 in the development of treatment resistance in CRC.

**Figure 4.**
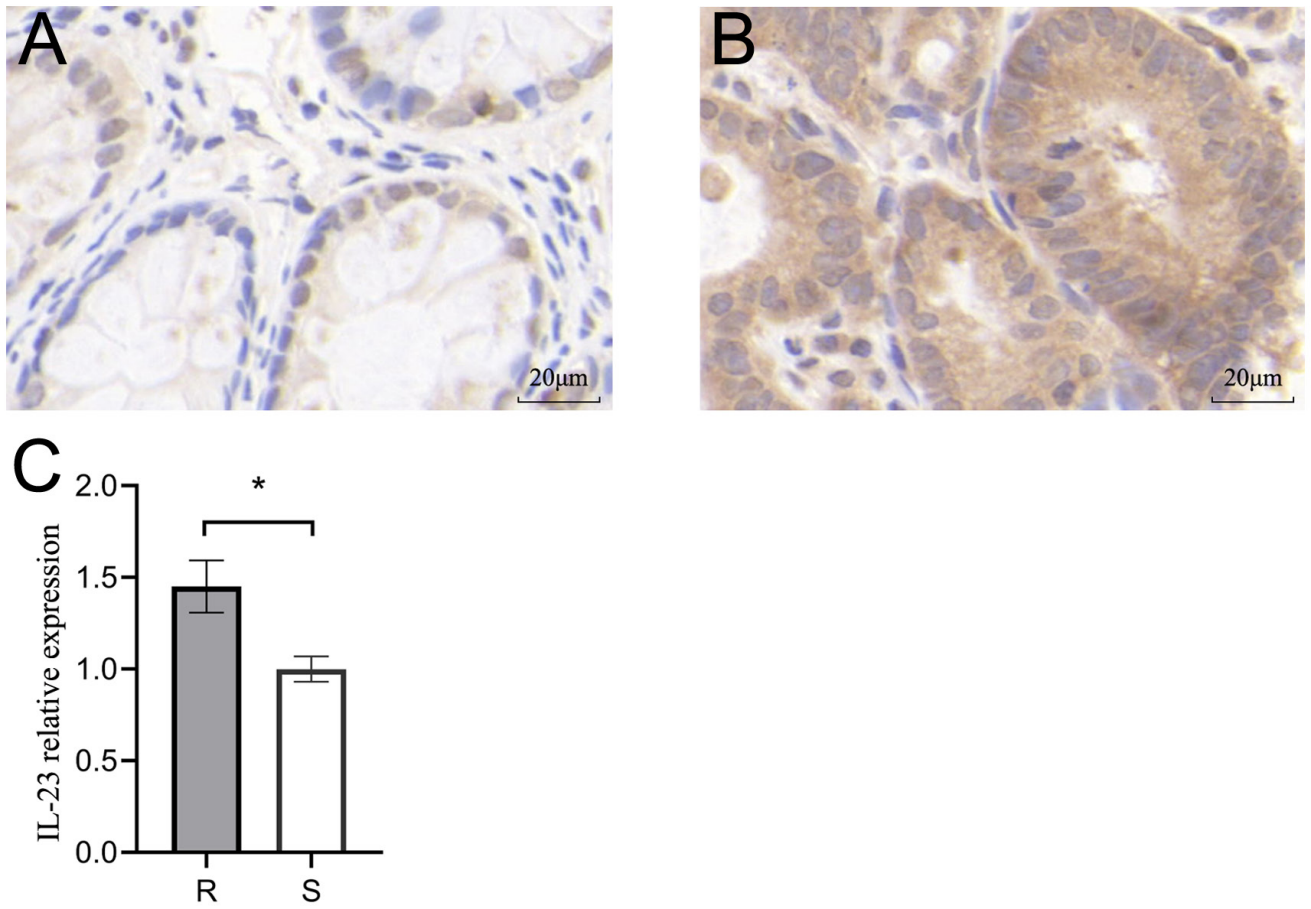
Immunohistochemical analysis of IL-23 expression in the 2 groups. (A) Expression of IL-23 in tumor tissues of the chemotherapy-resistant group; (B) expression of IL-23 in tumor tissues of the chemotherapy-sensitive group; and (C) statistical analysis of IL-23 expression in tumor tissues (R, chemotherapy-resistant group; S, chemotherapy-sensitive group. Magnification, 400×; * P < .05).

**Figure 5.**
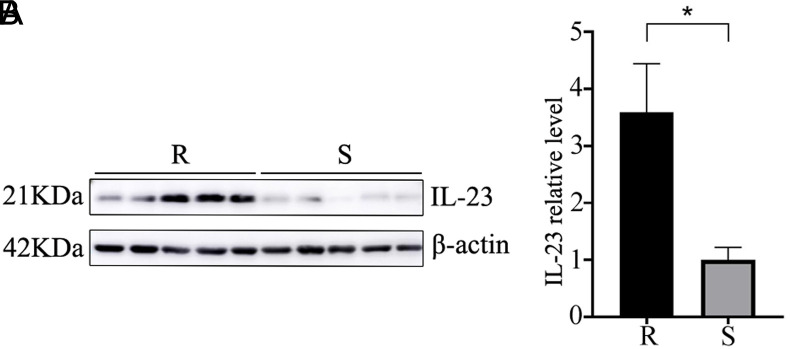
Expression of IL-23 in tumor tissues of the 2 groups, shown by Western blotting. (A) Images of blots; (B) quantification of gray-scale values (R, chemotherapy-resistant group; S, chemotherapy-sensitive group. *P < .05).

**Figure 6.**
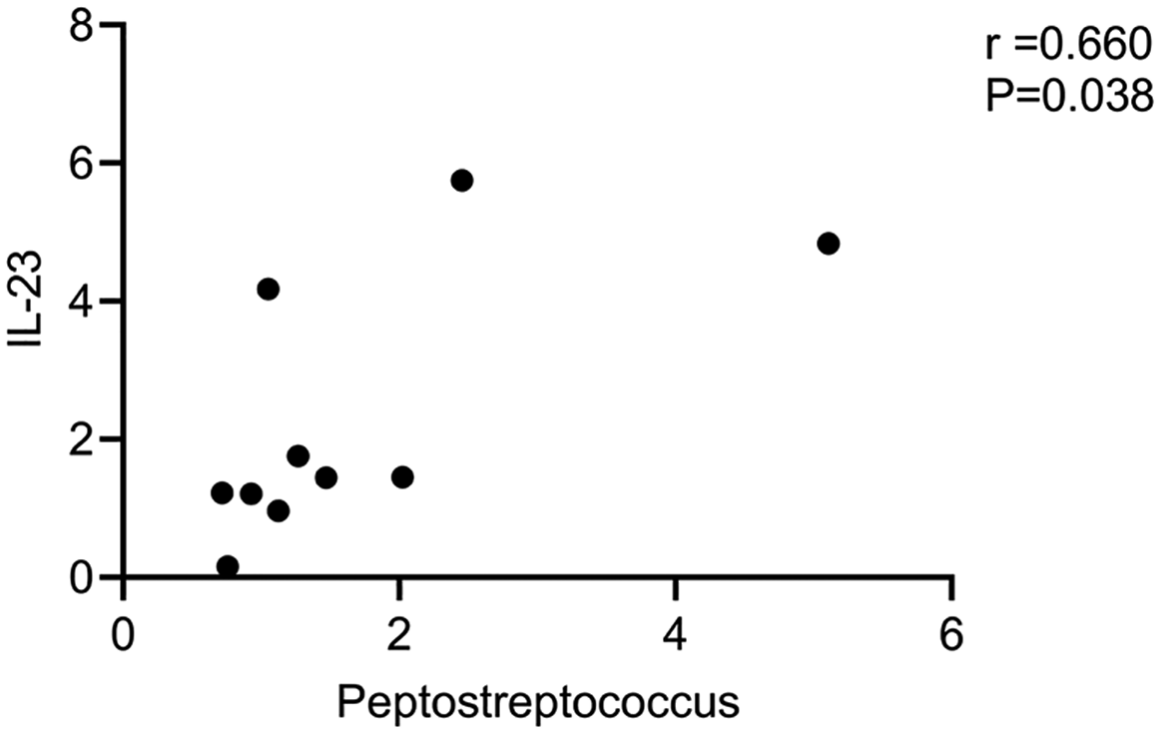
Correlation between *Peptostreptococcus* abundance and IL-23 expression.

A significantly greater abundance of *Peptostreptococcus* was observed in the intestines of patients in the chemotherapy-resistant group in this study, suggesting that these bacteria have a role in the development of chemoresistance in human CRC (Figure 3). In a comparison of high- and low-risk tumors, Weiting Ge and Hanguang Hu discovered that the former overexpressed IL-23A and had higher concentrations of CRC-related microorganisms, such as *Peptostreptococcus*. It has been shown that the products of bacterial invasion stimulate myeloid cells associated with tumors, leading to the release of IL-23.^11^ Many studies have demonstrated an association between IL-23 and CRC development. In this study, both Western blotting and IHC analyses revealed higher levels of IL-23 expression in tumors from chemoresistant CRC patients as compared to patients with chemosensitive disease, in line with prior findings (Figures 4C and 5B). This implies that IL-23 plays a role in the development of chemoresistance in human CRC. Our earlier research using both cellular and animal models indicated an association between *Peptostreptococcus* and CRC chemoresistance. The mechanism may involve recruitment of MDSCs. Using IL-23 stimulation of the EMT, the present study suggested that recruited MDSCs may enhance resistance to 5-Fu.^16^ Furthermore, we discovered a positive association between *Peptostreptococcus* abundance and IL-23 expression in CRC patients’ feces. It is thus suggested that IL-23 may contribute to the mechanism of chemoresistance in CRC, together with *Peptostreptococcus,* as shown in Figure 6 (*P* < .05). In summary, the findings of the study suggest that *Peptostreptococcus* is involved in chemotherapy resistance in human CRC, possibly through promoting increased expression of IL-23. These data offer strong support for the role of *Peptostreptococcus* in the onset of chemoresistant CRC.

Many studies to date have demonstrated the ability of *Peptostreptococcu*s to promote CRC tumor development, underscoring the clinical relevance of these bacteria such that they emerge as future targets for the treatment of CRC. In the present analyses, increases in intestinal *Peptostreptococcus* abundance in chemoresistant CRC patients were associated with the upregulation of IL-23. However, additional research will be necessary to clarify the mechanistic basis for these findings. Future multi-center clinical studies with larger sample sizes are needed to clarify the mechanisms through which *Peptostreptococcus* promotes chemoresistance. Cell- and animal-based research efforts have the potential to inform further therapeutic efforts, potentially providing improved benefits to CRC patients undergoing chemotherapeutic treatment.
